# Availability and adaption of exercise programs in pediatric oncology during the COVID-19 pandemic and beyond: a nationwide follow-up survey of providers in Germany

**DOI:** 10.3389/fped.2024.1372261

**Published:** 2024-03-22

**Authors:** Sabine Kesting, Dominik Gaser, Jennifer Queisser, Miriam Götte, Irene von Luettichau, Christiane Peters, Renate Oberhoffer-Fritz, Gabriele Gauß

**Affiliations:** ^1^ Department of Pediatrics and Children's Cancer Research Centre, Kinderklinik München Schwabing, TUM School of Medicine and Health, Technical University of Munich, Munich, Germany; ^2^ Department Health and Sport Sciences, Institute of Preventive Pediatrics, TUM School of Medicine and Health, Technical University of Munich, Munich, Germany; ^3^ Children’s Oncology Network Bavaria, KioNet, Erlangen, Germany; ^4^ Clinic of Pediatrics III, Hematology and Oncology, University Hospital Essen, Essen, Germany; ^5^ West German Cancer Center Essen, University Hospital Essen, Essen, Germany

**Keywords:** childhood cancer, physical activity, exercise program, COVID-19, survey, restrictions, adaptions

## Abstract

**Background:**

The COVID-19 pandemic has presented major challenges to clinical practice and delivery of care programs throughout all health care systems. Exercise programs, that are implemented in most centers for pediatric oncology in Germany, are a relatively new care program however with high clinical impact and health benefits.

**Objective:**

The impact and consequences of the pandemic on the delivery and availability of exercise programs in Germany for pediatric cancer patients and survivors are unknown. A national survey analyzed restrictions, challenges and novel approaches of exercise program delivery and scientific research.

**Method:**

A two-stage online survey was distributed to providers of exercise programs (acute clinics, non-clinical institutions, rehabilitation facilities) via the established Network ActiveOncoKids. Data was collected during the pandemic in 2022 and 2023 using a combination of open and closed questions.

**Results:**

In total, *n* = 27 (response rate: 82%) and *n* = 17 (response rate: 63%) providers participated in the first and second survey, respectively. Findings pointed out restrictions in 85% of all exercise programs in 2020 and 2021, with slight reductions in 2022. During pandemic, restrictions with major impact arose within exercise offers during follow-up and declined gradually. Whereas restrictions within the setting of acute therapy had medium or minor impact but persisted beyond. Delivery of provided exercise programs necessitated adaptions, including digital methods, supervised interventions from a distance and change of locations.

**Discussion:**

The findings highlight the adaptability, the demand and the potential of exercise programs in pediatric oncology. We assume that exercise professionals have used the pandemic-related challenges to review and modify existing concepts and made adaptations according to local conditions and novel tools for the provision of exercise programs. Nevertheless, a conspicuous lack of exercise-related care has become evident in certain patients and survivors. Further expansion of programs is imperative to address and accommodate all pertinent needs.

## 1 Introduction

Physical activity and exercise are essential for a healthy physical, psychological and social development during childhood (1, 2). Therefore, a physically active lifestyle should not only be promoted for healthy children and adolescents (3), but especially for children with chronic diseases and impairments (4, 5). Childhood cancer is a rare disease with a yearly number of 2,200 new cases (0–18 years) in Germany (6) and a worldwide incidence of 15.6 per 100,000 children (7). Intense treatment regimens led to 5-year survival rates above 80% (8), but this life-threatening disease often results in late sequelae (9). Physical activity and engagement in exercise are frequently low during (10) and following treatment (11). However, evidence is growing on positive effects of therapy-adjuvant, supervised exercise interventions (12–14) and approaches to increase physical activity in survivors of childhood cancer (15–17) worldwide within the last 15 years. The need to implement exercise programs as usual care led to the development of a nationwide network in Germany: The Network ActiveOncoKids (NAOK) (18). Recent consensus-based recommendations from the multidisciplinary NAOK highlight the importance of providing movement and exercise within this vulnerable group of patients and survivors (19).

The COVID-19 pandemic, beginning in 2020, has highly affected everyday life in children and adolescents. Worldwide, government instructions including social distancing, closing of schools and sport clubs deprived children and adolescents of most kinds of movement experiences. This situation resulted in decreased physical activity patterns (20). Moreover, mental health status of both patients and their parents was negatively affected during the onset of the COVID-19 pandemic (21). The pandemic also had a global impact on childhood cancer outcomes and care delivery (22). Apart from obviously indispensable medical treatment of childhood cancer, supportive care programs, including psychosocial support, school education and programs to support physical activity and exercise, have been impaired, interrupted or even stopped during the pandemic status in Germany. Clinical practice in childhood cancer care has been challenged due to government regulations, risk factors and strict hygiene directives (23). Short-termed adaptions and practical solutions seemed to be necessary to maintain delivery of the widespread status of exercise programs in Germany compared to the global situation (24).

To the best of our knowledge, the results of this survey represent the initial attempt to offer a comprehensive overview of the landscape of exercise programs in pediatric oncology during the COVID-19 pandemic in Germany. Furthermore, we provide a thorough synthesis and discussion of limitations inherent in exercise programs and the challenges they present. Finally, newly formulated approaches designed to prospectively broaden outreach are illustrated.

## 2 Methods

### 2.1 Procedure

The survey was performed in two steps. From January 19th until February 27th in 2022 (Survey 1.0) and as a follow-up from January 18th until February 28th in 2023 (Survey 2.0). Both retrospective surveys were conducted in cooperation with the NAOK (18) in Germany concerning methodology and recruitment. Within the NAOK, the majority of existing pediatric oncology exercise programs providers nationwide are connected. In total, 36 sites and providers with specific exercise programs out of 60 acute clinics in Germany are currently represented in the NAOK in 2024.

### 2.2 Eligible providers

For the first survey, the NAOK headquarter at the University Hospital Essen distributed the link for the online questionnaire to all NAOK member sites hosting an exercise program with or without scientific studies. Every site, including in- and outpatient clinics, rehabilitation facilities and non-clinical institutions, was invited for participation via e-mail (*n* = 33). One reminder was sent one week before closing of data collection to maximize the response rate. Each centre was asked to select one person to answer the questionnaire (ideally the person responsible for the exercise program) to avoid multiple responses per centre.

For the follow-up survey in 2023, all participants from the first survey were contacted again and invited to complete the second online questionnaire (*n* = 27). One reminder was sent one week before closing of data collection to maximize the response rate.

### 2.3 Data collection and analysis

The questions were collaboratively developed by researchers from the Technical University of Munich (TUM), Institute of Preventive Pediatrics, Department Health and Sport Sciences, Children's Hospital Munich Schwabing, Children's Cancer Research Centre, Department Clinical Medicine, TUM School of Medicine and Health and the NAO headquarter, University Hospital Essen. This survey was hosted using an online survey tool (LimeSurvey, LLC), chosen because of its compatibility with all terminal devices and compliance with data protection regulations.

All respondents provided consent to participate voluntarily via check-boxes within the online survey tool, confirming they understood the aim of this survey and were providing information on behalf of their centre. The voluntary option was given to provide the respondent's name, contact data for queries and the location of the participating centre.

The online questionnaire was internally tested by academic colleagues for readability and ease of use including accessibility from any device and verified for technically flawless handling prior to dissemination to respondents. A mixed methods approach was used, including quantitative and qualitative data collection (free-text responses). The duration to complete the questionnaires was approximately 15 min each. All results are presented as frequency distribution and free-text responses are provided categorized.

### 2.4 Questionnaires

The online questionnaire comprised the following categories: (A) general questions, (B) impact of the COVID-19 pandemic on exercise programs [within different phases of treatment (acute, outpatient, follow-up) due to restrictions], (C) approaches to maintain exercise programs, (D) challenges of COVID-19 pandemic regarding the delivery of exercise programs, (E) impact on scientific research, (F) feedback/opinions.

Within the initial Survey 1.0, a major part of the questions was specifically related to the impact of COVID-19 on the availability of exercise programs and the conduction of scientific studies (see Supplementary Table S1, supplementary material). Questions regarding restrictions considered individual pandemic waves with very high COVID-19 incidence in Germany (25). The follow-up Survey 2.0 focused on the continuous availability of exercise programs, further developments, adaptions to enable access to those programs for childhood cancer patients and survivors and novel approaches for future integration (see Supplementary Table S2, supplementary material). Adaptions are considered as changes in procedures to continue programs taking pandemic-related circumstances into account (e.g., individual sessions instead of group sessions, reduction of exercise intensity due to aggravated breathing wearing face masks). Novel approaches are delineated as actions and ideas that remained untapped before the pandemic but have since been adopted and persistently continued after their successful implementation (e.g., digital offers).

## 3 Results

The following paragraphs present results from both Survey 1.0 and the follow-up Survey 2.0. With regard to associations and distinctions, results are compared in-between both surveys (participants, restrictions, adaptions and approaches) and subdivided according to different questions in Surveys 1.0 and 2.0 (scientific research, challenges, feedback).

### 3.1 Participants

**Figure 1:**
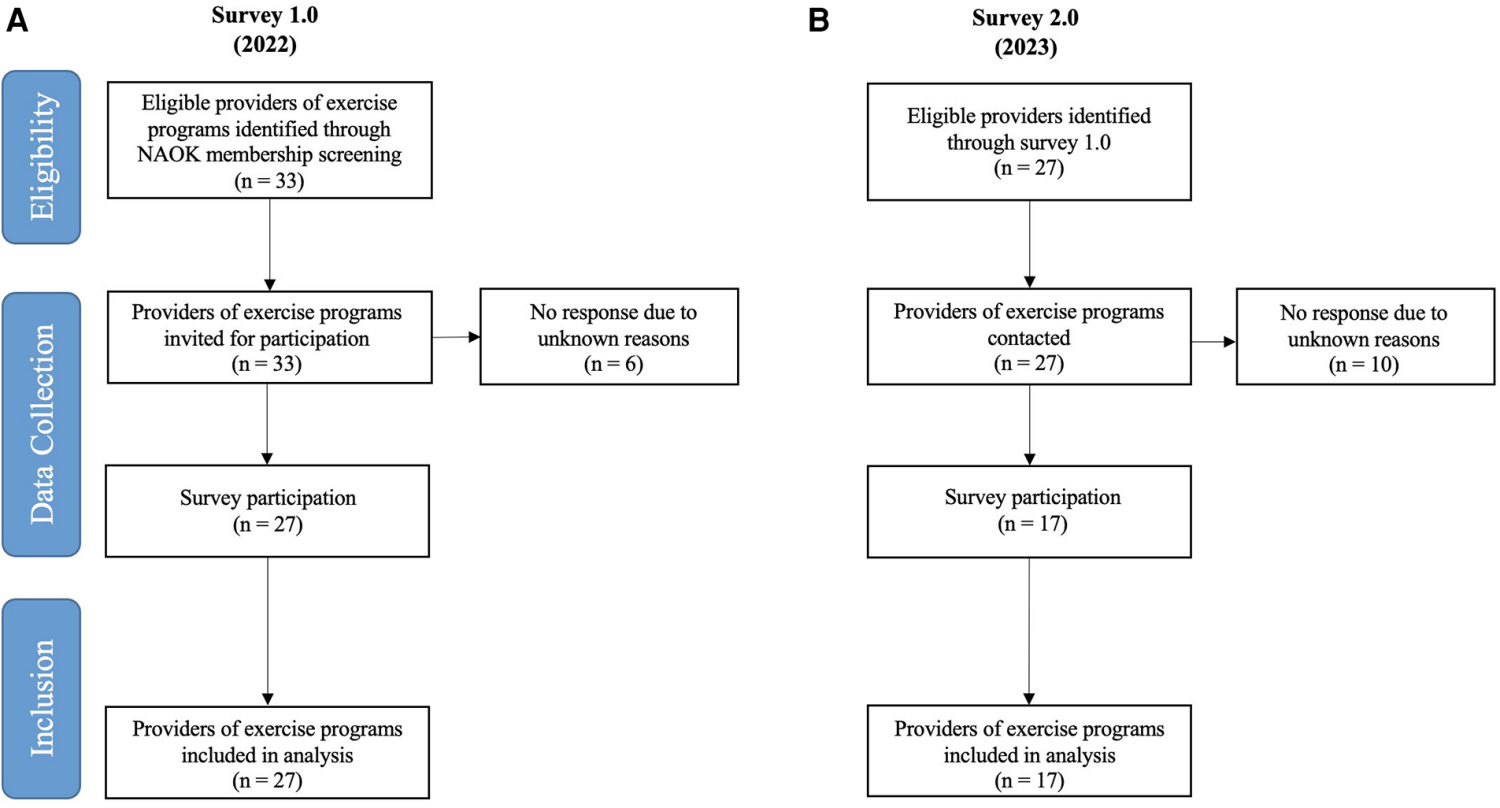
Flow chart of (**A**) the baseline Survey 1.0 (2022) and (**B**) the follow-up Survey 2.0 (2023). N, number.

Eligibility and participation data regarding both surveys are presented in the flow chart (see Figure 1). In Survey 1.0, out of the 33 eligible providers, 27 completed the questionnaire, culminating in a response rate of 82%. For the follow-up survey (Survey 2.0), out of a total of 27 contacted providers, 17 responded, yielding a response rate of 63%. Reasons for non-participation in both surveys remain unknown. Only in Survey 1.0, one in-patient rehabilitation facility answered the questionnaire.

Table 1 shows the overview of providers participating in both surveys. To prevent misunderstanding of the following results, it needs to be stated that some centers (Survey 1.0 *n* = 12, Survey 2.0 *n* = 9) provide offers throughout all settings and phases of treatment (acute and maintenance therapy, follow-up care). Therefore, the addition of results does not necessarily add up to 100%.

**Table 1:** Characteristics of participating institutions within Survey 1.0 (2022) and Survey 2.0 (2023).

Characteristics	Survey 1.0 *N* (%)	Survey 2.0 *N* (%)
Eligible providers	33 (100%)	27 (100%)
Providers with response	27 (82%)	17 (63%)
Setting	*N* = 27	*N* = 17
Acute therapy	20 (74%)	12 (44%)
Maintenance therapy	16 (59%)	11 (41%)
Follow-up care	19 (70%)	12 (44%)
Institution	*N* = 27	*N* = 17
Acute clinic (in- and outpatient)	16 (59%)	14 (52%)
Rehabilitation facilities	3 (11%)	1 (4%)
Non-clinical institutions/research institutions/university/activity camps	3 (11%)	1 (4%)
Consulting service^a^	1 (4%)	1 (4%)
Not specified	4 (15%)	0 (0%)

^a^ Network ActiveOncoKids consulting service.

### 3.2 Impact on exercise programs

**Figure 2:**
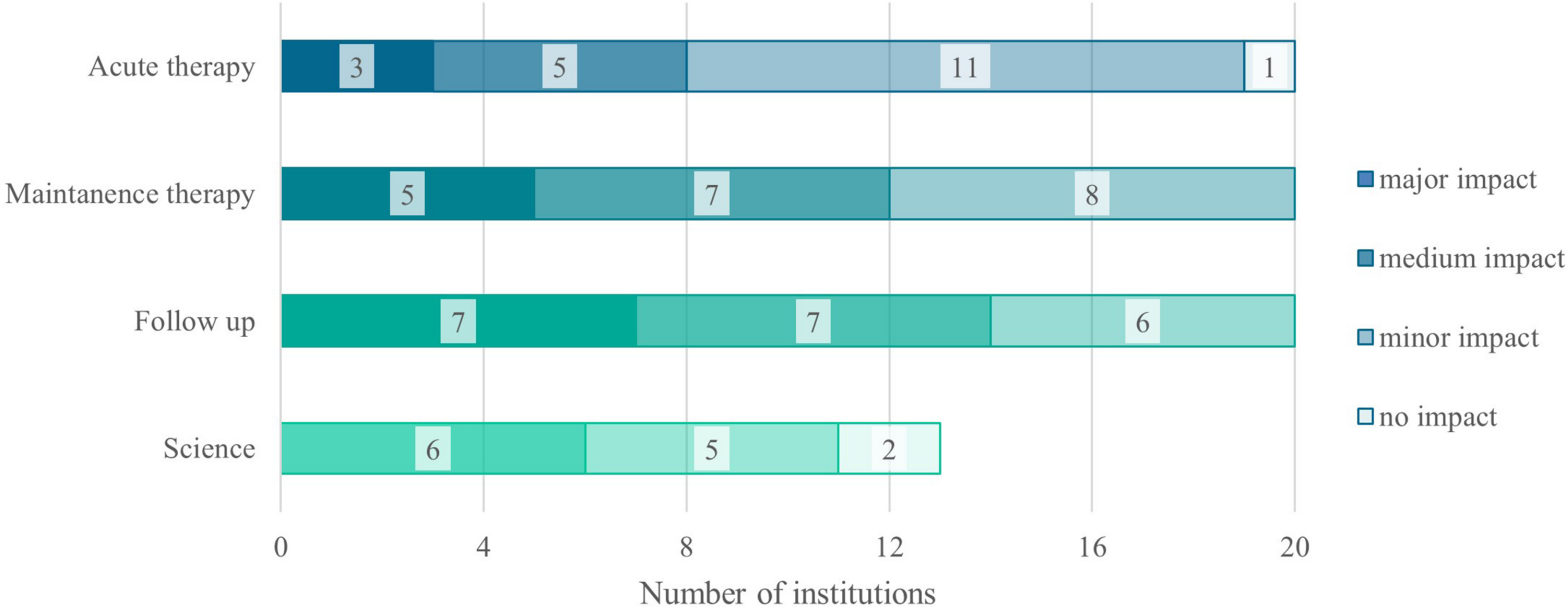
The pandemic's impact on individual parts of exercise programs within Survey 1.0 (2022). N, number; *n *= 13/27 conducted any scientific studies.

Within Survey 1.0, data regarding the pandemic's impact on individual parts of exercise programs was collected (see Figure 2). The most frequently reported response was major impact during follow-up and maintenance therapy. Minor impact was estimated most often during acute therapy. Scientific research was majorly affected in some centers, whereas all respondents who were not conducting studies did not reply to this issue.

### 3.3 Restrictions

**Figure 3:**
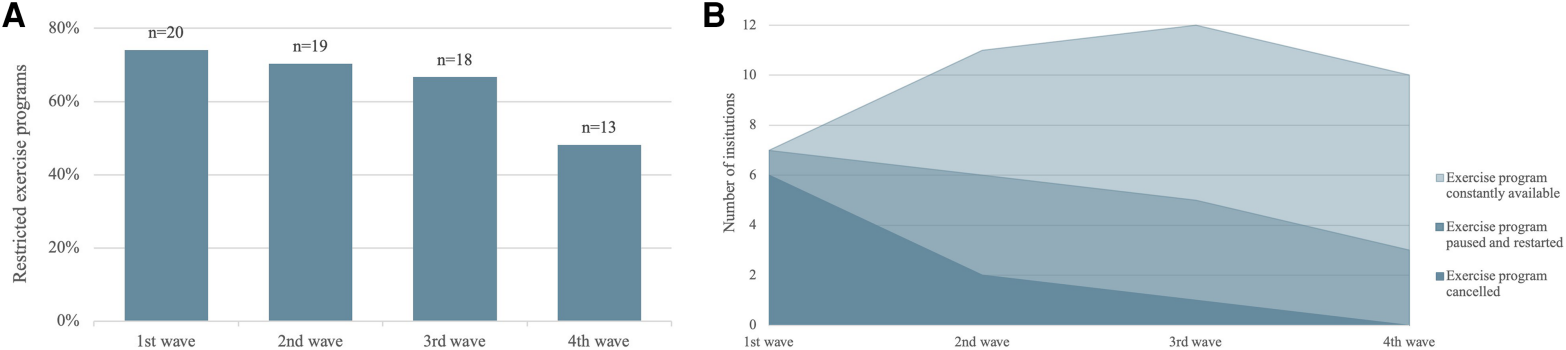
(**A**) restrictions on and (**B**) status of exercise programs considering pandemic waves (03/2020–02/2022). Pandemic waves are defined according to the rates of high infection reported by the German public health institute: 1st wave March–May 2020, 2nd wave October 2020–February 2021, 3rd wave March 2021–June 2021, 4th wave August 2021–February 2022 (25). Numbers of institutions in (**B**) are not stacked with regard to the status of exercise programs, but are overlapping and amount to the number of each wave shown in (**A**).

In Survey 1.0, the majority of respondents stated restriction due to the pandemic (85%) in 2022. Most severe restrictions were mentioned during the first wave at the beginning of COVID-19 in 2020 with a slight reduction in the second and third wave. In the fourth wave in 2022, restrictions concerning exercise programs regressed according to the respondents (see Figure 3A).

With the exception of the first wave, the majority of exercise programs remained consistently available. Instances of cancellations and pauses, followed by program restarts, diminished across subsequent waves. Notably, no programs were canceled during the fourth wave (see Figure 3B).

In addition to numerous restrictions, a total of *n* = 6 exercise programs was established newly amid the pandemic context during the initial two years of COVID-19 (2020–2021), while *n* = 21 programs were pre-existing.

In Survey 2.0, the status of exercise programs was not yet comparable to a pre-pandemic level in 2023. However, restrictions declined noticeably. The most incisions were stated to consist during acute therapy with 58%, followed by maintenance therapy with 62% compared to the status before the COVID-19 pandemic. Exercise programs during follow-up care reached 80% of the pre-pandemic level and consulting service (*n* = 1) was unrestricted.

In Survey 2.0, the constraints identified in Survey 1.0 were systematically examined, with a particular emphasis on the perceived impact of these restrictions across various settings. In hindsight, participants predominantly attributed a substantial impact on exercise programs to institutional and governmental restrictions. Conversely, other aspects related to concerns about infections received ratings indicating medium impact. Perceived impact on the organization of exercise programs was rated low. Participants indicated a comparable level of impact across all settings.

### 3.4 Scientific research

In Survey 1.0, the pandemic's influence on the examination of scientific studies encompassed both their initiation and subsequent disruption or cessation. Notably, one center had to suspend a study involving high aerosol emissions for four weeks. Among the ongoing studies (*n* = 14), adherence to stringent hygiene protocols was paramount, with measures such as the use of face masks (*n* = 13), mandatory negative COVID-19 tests (*n* = 4), and the implementation of individual training sessions (*n* = 10). One center reported heightened operational efforts attributed to limited space and challenges associated with equipment transport. Furthermore, adaptations were made in response to logistical constraints, with one center transitioning elements of study interventions and testing to an online format. Free-text responses conveyed instances of postponed or canceled study visits due to isolation regulations, impediments arising from the suspension of human studies at the local university, the need for special authorization to proceed with studies, and stringent entrance requirements for external scientific personnel.

### 3.5 Adaptions and novel approaches

Free-text responses to all questions related to adaptions of practice and novel approaches (defined in the methods paragraph) in exercise programs according to specific restrictions within the first two years of the pandemic highlighted a number of common themes. The major aspect was reduced contact between individuals to limit the risk of infection. On the one hand, contact was limited to sessions within the clinics and without contact to others than patients and exercise physiologists. This led to cessation of offers by external personnel, no participation of friends and siblings and individual sessions instead of group sessions were realized. On the other hand, exercise sessions were performed according to strict hygiene measures (e.g., face mask, intensified disinfection) and careful selection of equipment and locations. During follow-up care, offers within groups were limited to a small number of participants, and some programs had to be interrupted during waves of high infection rates.

Additionally, novel approaches were tested and used as a solution to counteract barriers resulting from regulations and restrictions. Live online offers and on-demand videos produced by diverse sites within NAOK were named to provide ideas for maintaining physical activity and exercise at home and during in-patient stays. Those approaches were either used at individual centers and on the NAOK YouTube Channel at the homepage (26), accessible for all German-speaking countries. During treatment as well as during follow-up care programs, centers integrated outdoor sessions to continue exercise interventions while reducing the risk of infection within groups following the government's behavior rules. To increase physical activity during home stays, individual training schedules were prepared for patients and survivors. To provide options for advising and addressing questions regarding physical activity and exercise without necessarily visiting the clinics, consulting service hours were offered via telephone and online tools. During in-patient stays, especially during periods of isolation, training equipment was borrowed, including clear instructions for use. One center started to prepare and distribute cinch bags filled with exercise equipment, e.g., resistant bands, balloons, training manual, and QR codes for on-demand training videos by NAOK during acute therapy.

### 3.6 Feedback to adaptions and novel approaches

The feedback received indicated a moderate level of satisfaction, with participants and families expressing contentment in engaging with a variety of exercise interventions and offerings, according to the survey responses. Conversely, critical feedback highlighted regrets pertaining to individual sessions vis-à-vis group formats, a perceived elevation in the value of in-person events compared to virtual counterparts, and spatial limitations within the in-patient setting due to restricted range of motion. Furthermore, an acknowledgment of heightened screen time, exacerbated by online training sessions in conjunction with existing online schooling commitments, was underscored.

**Figure 4:**
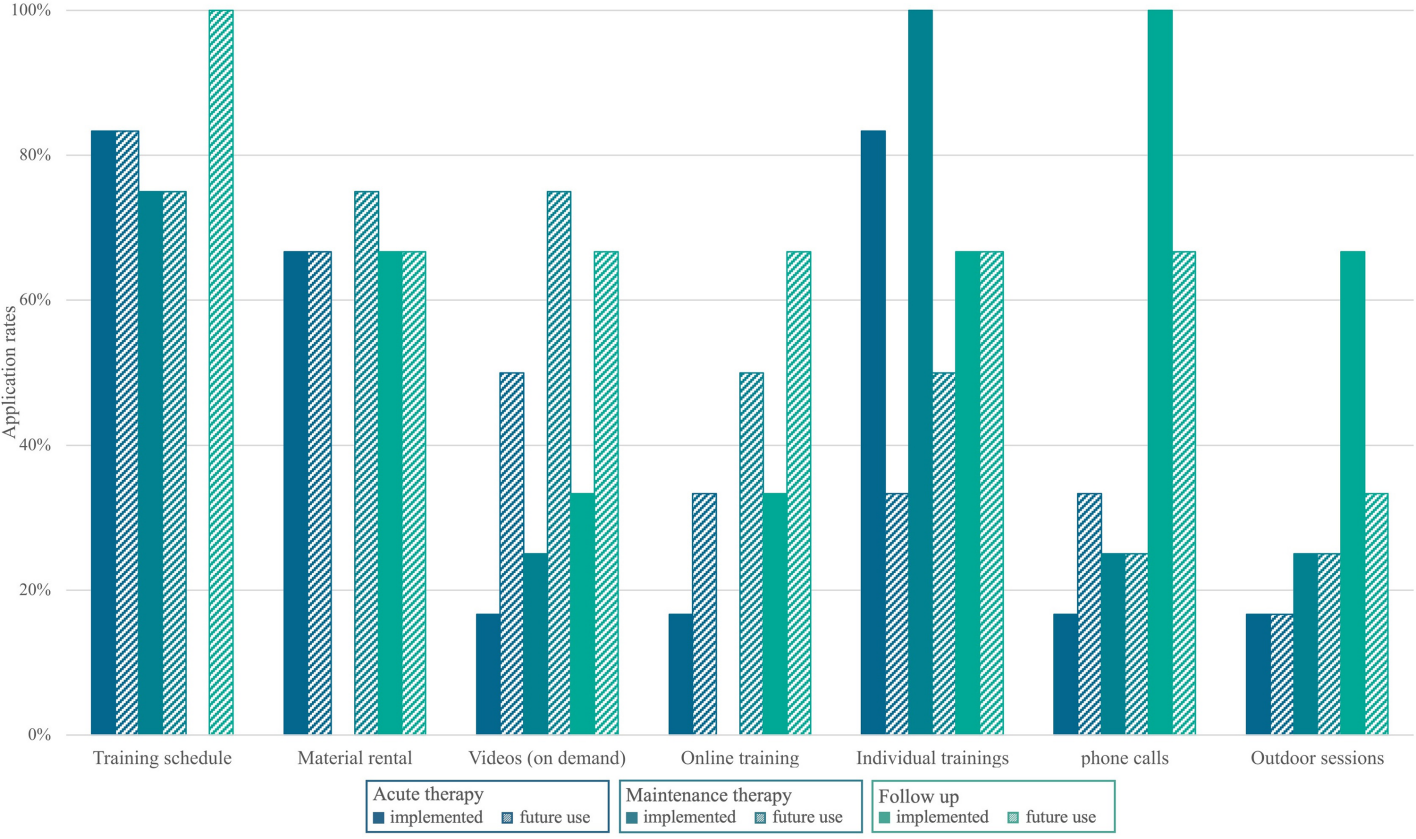
Novel approaches developed and applied during the pandemic addressed within Survey 2.0 (2023), including future perspectives of use. This question was answered by *n* = 6 centers. Clear bars indicate approaches already implemented in exercise programs. Shaded bars indicate the expected future use and potential of novel approaches.

Figure 4 summarizes novel approaches and different uses in several settings. Six respondents answered this question, whereas eleven did not provide any response.

### 3.7 Acceptance and feedback of novel approaches by patients and survivors

Acceptance and feedback by patients, survivors and families were reported by the perspective of the participants filling in the questionnaire. According to this, reactions regarding novel approaches were mixed. Online offers were only accepted partly, whereas workout plans for the use at home were wished for more frequently without information about the actual execution, in general. Telephone consultation had already been implemented before and no difference became obvious during the pandemic. Outdoor training was particularly well-received, especially in the context of follow-up care.

### 3.8 Challenges

During the COVID-19 pandemic, obstacles to maintaining exercise programs intensified. Survey participants emphasize increased barriers to build personal relationships and trust due to isolation (during patients' COVID-19 infection) and face masks. Additionally, lack of personnel in all areas of care due to infections was named, leading to an increased workload for all individuals and emotional stress due to a strained working atmosphere. Especially in exercise programs, the demand was often stated high due to cancelation of mainly all other offers (school, arts therapy, music therapy, occupational offers by external personnel) during the first and second wave. Furthermore, the conditions for presenting offers were deemed formidable: constrained space, heightened time requirements owing to regulations and hygiene protocols, a demand for remarkably adaptable responses, and the potential deterrent effect of face masks on certain patients, given the associated risks of headaches, premature fatigue, and impediments to effective communication.

Furthermore, centers emphasize a reduction in funding due to the cancellation of charity events, emphasizing the imperative to explore alternative financial support for program provision. Additionally, the absence of events and opportunities for professional networking and experience exchange was identified as a challenge, particularly impacting newcomers in the field of pediatric exercise oncology. Lastly, delays in personnel recruitment attributed to regulatory constraints and restrictions were also noted.

### 3.9 Positive aspects of the pandemic

However, some positive aspects were extracted as well. The extend of offers to digital tools resulting in increased flexibility and outreach was rated positive. Furthermore, according to the respondents, concerns regarding online tools decreased during the pandemic period and home training programs supervised online were implemented. Additionally, a strong commitment and appreciation of medical personnel regarding exercise programs was named. Overall, respondents emphasized an increased motivation to enhance, restructure, and professionalize existing programs. Within patients, survivors and parents, according to the respondents physical activity and exercise seemed to attract more attention, higher demand and acceptance. They also noted an increase of public appreciation of exercise programs within the general population.

## 4 Discussion

To our knowledge, this is the first evaluation of how the COVID-19 pandemic has impacted the availability of exercise programs in pediatric oncology. This survey comprised a baseline survey in 2022 (Survey 1.0, covering the period since the beginning of the COVID-19 pandemic) and a follow-up survey in 2023 (Survey 2.0). Considering changes in behavioral patterns and reduced physical activity in the population of healthy children and adolescents due to COVID-19 pandemic (20), support and required offers for children and adolescents with chronic diseases and health impairments become obvious.

The principal positive observation revealed the emphasis on the significance and, consequently, the maintenance or early resumption of paused exercise programs. Despite the heightened sensitivity observed within this patient cohort, the deemed importance of these programs warranted their continuation. This reaffirms prior findings attesting to the safety and feasibility of supervised exercise programs (12, 27, 28). Furthermore, it was clear that there was a significant and even increasing demand, especially during a period of restrictions. Despite challenges, solutions were found, and new approaches were implemented. In addition, six exercise programs were successfully introduced during the COVID-19 pandemic. Scientific studies mostly continued, aiming to fill research gaps (29, 30).

The results can be discussed on three different levels and associated key factors: (1) professional level, (2) patient level, and (3) social level.

Navigating exercise programs during the COVID-19 pandemic presented persistent challenges on the professional level. On-site observations were rendered impossible, underscoring the importance of a robust network and experience. The absence of charity events exacerbated funding issues, emphasizing the need for diverse financial support. Ongoing restrictions, particularly during acute therapy, posed sensitivity issues, but a noteworthy reduction of restrictions during certain waves suggests a learning curve and adaptability gained over time. In summary, while challenges persist, adaptability and insights gained during the pandemic offer avenues for improvement in managing exercise programs effectively. At the patient level, the tailored design of exercise programs, the development of online offerings, and increased flexibility are motivating factors. The expansion of phone consultations, creation of personalized training schedules, improved reachability, and provision of options for continued participation further underscore the compelling rationale for the comprehensive restructuring, optimization, and professionalization of these programs. This motivation indicates a collective commitment to enhancing the efficacy and accessibility of exercise interventions at the patient level, demonstrating a proactive response to evolving healthcare needs.

The impact on the social level is noteworthy, as exercise programs have been implemented and conducted despite substantial barriers. The resilience in overcoming challenges speaks to a collective commitment and determination within healthcare teams. The heightened attention and visibility garnered by these efforts underscore the recognition of the importance of exercise programs in the broader healthcare context (31, 32). The collective commitment at the social and team levels suggests the potential for ongoing improvements in integrating and optimizing of exercise programs within healthcare practices.

In relation to novel approaches, only six out of 17 survey participants in Survey 2.0 responded to these inquiries. While this comprises just one-third of the respondents, it necessitates careful examination. Amid the COVID-19 pandemic, six programs were freshly implemented, and they may have already incorporated innovative methods, such as digital offerings. The consultation service demonstrated a higher inclination toward digital interactions than to face-to-face exercise offerings. It could be inferred that most exercise programs in Germany had already achieved high standards in the years preceding the pandemic (18, 19), rendering novel approaches less imperative. Moreover, throughout the pandemic, staff members were predominantly engaged in delivering existing exercise programs, and resources to explore novel approaches were constrained due to time-consuming restrictions. Nonetheless, some forward-thinking strategies were adopted during the pandemic, including: (1) the introduction of digital and resource-efficient offerings to broaden outreach, (2) the implementation of training schedules to empower patients' autonomy and facilitate exercise during home stays, and (3) the provision of material rentals to enhance exercise options for in-patient stays on weekends. Despite these options, the provision of clear instructions on how to utilize these offerings and materials is crucial to empower patients to engage in physical activity autonomously. Further use and potential of novel approaches and adaptions was considered even higher than currently used. Nevertheless, the anticipated future potential of certain novel approaches (Figure 4) was deemed to be lower compared to their current utilization, particularly in the case of individual training sessions and outdoor training. This observation may indicate the prevailing landscape in pediatric oncology, wherein individual sessions are the conventional norm, and there exists a substantial preference for group sessions during follow-up care. While outdoor sessions may find integration in the future, their prominence during the COVID-19 era may have contributed to a perception that does not necessarily position them as outstanding in the long-term context. These approaches align with the evolving landscape of healthcare, emphasizing patient-centered approaches and the integration of technology for improved accessibility and engagement also explored in adult exercise oncology (33) and other chronic diseases in childhood (34).

## 5 Limitations

Limitations in our study warrant consideration for a more nuanced discussion. Firstly, there is a potential for bias in the representation of providers, especially if particular programs, such as NAOK, exert a dominant influence. Further exploration into non-respondents’ reasons and status are essential for a comprehensive understanding. Secondly, the findings might not be universally transferable to countries with fewer programs. Thirdly, the completion of questionnaires was limited to exercise professionals exclusively, with the exclusion of input from other professional perspectives. It is acknowledged that incorporating opinions from diverse professional backgrounds could have broadened the scope and depth of the survey. However, they can still serve as a valuable role model, offering new ideas for implementation and adaptation in diverse contexts.

## 6 Conclusion

This survey unveiled the implementation of innovative approaches to actualize exercise programs during periods of pandemic-related restrictions. However, it appears that exercise programs in Germany are already held to rigorous standards. These results of exercise programs being maintained during difficult situations, facing restrictions, and short-dated successful adaptions might encourage other countries to initiate similar programs for pediatric cancer patients and survivors. Additionally, the expansion of digital offerings has the potential to enhance participants' overall access to exercise programs and broaden their reach. These surveys suggest a robust commitment and appreciation for the exercise programs in pediatric oncology.

## Data Availability

The raw data supporting the conclusions of this article will be made available by the authors, without undue reservation.
