# Supplementary material for: Availability and adaption of exercise programs in pediatric oncology during the COVID-19 pandemic and beyond: a nationwide follow-up survey of providers in Germany
Source: Front Pediatr. 2024 Mar 22;12:1372261. doi: 10.3389/fped.2024.1372261 (PMC10995395; doi:10.3389/fped.2024.1372261)
Supplement: Supplementary file 1 [file Table1.pdf]

Supplementary Material

Availability and adaption of exercise programs in pediatric oncology during the COVID-19 pandemic and beyond: A nationwide follow-up survey of providers in Germany

Sabine Kesting[1,2,3], Dominik Gaser[1,2,3], Jennifer Queisser[1,2], Miriam Götte[4,5], Irene von Luettichau[1,3], Christiane Peters[2], Renate Oberhoffer-Fritz[2], Gabriele Gauß[4*]

[1] Department of Pediatrics and Children's Cancer Research Centre, Kinderklinik München Schwabing, TUM School of Medicine and Health, Technical University of Munich, Munich, Germany

[2] Department Health and Sport Sciences, Institute of Preventive Pediatrics, TUM School of Medicine and Health, Technical University of Munich, Munich, Germany

[3] Children's Oncology Network Bavaria, KioNet, Erlangen, Germany

[4]Clinic of Pediatrics III, Hematology and Oncology, University Hospital Essen, Essen, Germany

[5]West German Cancer Center Essen, University Hospital Essen, Essen, Germany

***Correspondence:** Gabriele Gauß**:** Gabriele.Gauss@uk-essen.de

TABLE S1 Closed and open questions within Survey 1.0 (2022).

**Q1**	**In which phase during childhood anti-cancer treatment are you offering an exercise program and/or conducting scientific studies?** *(multiple answers possible)*						
	Acute in-hospital therapy	Maintenance therapy	Follow-up care in the hospital	Follow-up care outside the hospital	In-patient rehabilitation facility	Other	
**Q2**	**Did your exercise program already exist prior to COVID-19 pandemic in March 2020?**						
	Yes	No, the program was implemented during COVID-19 pandemic					
**Q3**	**How much did the COVID-19 pandemic affect the following areas of the exercise program?** Exercise programs during acute therapy/during maintenance therapy/during follow-up care and scientific studies						
	Very strong impact	Strong impact	Low impact	No Impact	Not applicable		
**Q4**	**Did any restrictions occur during COVID-19 pandemic regarding the exercise program?**						
	Yes	No					
**Q5**	**During which moment in time did restrictions occur regarding the exercise program?** *(consideration of pandemic waves with a high incidence; multiple answers possible)*						
	1st wave/1st lockdown March-May 2020	2nd wave/2nd lockdown October-February 2021	3rd wave March-June 2021	4th wave August 2021- February 2022			
**Q6**	**Did you need to interrupt or stop the exercise program?** *(question for each wave and setting separately)*						
	No, we neither interrupted nor stopped.	Yes, we interrupted and continued.	Yes, we stopped the program.				
	**How long was the duration of the interruption?** *(question for wave and setting)*						
	Less than 4 weeks	Between 4 weeks and 3 months	Between 3 and 6 months	More than 6 months			
	**Which restrictions occurred?** *(multiple answers possible)*						
	Cessations of programs by internal staff.	Cessations of programs by external staff.	Only individual training was allowed.	Program was only allowed at selected locations.	Program was only allowed with limited selection of equipment.	Program was allowed with strict hygiene measures.	Inclusion of siblings and friends was not possible.
	Barriers for integration into sport structures due to missing offers	COVID-testing procedure delayed or hampered the program	Exercise only possible for study participants	Limited number of participants in group offers	No contact sports	Other	
**Q7**	**Which approaches were used or implemented to maintain the exercise program during COVID-19 pandemic?** *(multiple answers possible)*						
	Online offers (video training or instructions *via* video)	Training schedule	Consultant hours *via* telephone	Outdoor training	Strict division of oncological participants within group offers		
	**How did the target group adopt these new approaches?** *(question for each approach separately)*						
	Very good	Good	Partly	Bad	Very bad	Other	
	**Please add any further approaches that have not been named previously and describe how they have been adopted.** *(optional)*						
	Free-text answers						
	**How was the general feedback regarding those newly implemented approaches by the participants?**						
	Positive	Rather positive	Partly	Rather negative	Negative	I am not aware of any feedback.	
	**Was there any negative feedback regarding those newly implemented approaches?** *(optional)*						
	Free-text response						
	**Was there any positive feedback regarding those newly implemented approaches?** *(optional)*						
	Free-text response						
**Q8**	**Did the COVID-19 pandemic bring concrete challenges regarding the conduction of exercise programs? Please name them.**						
	Free-text response						
**Q9**	**Did you have to interrupt or stop scientific studies?**						
	Yes, studies were stopped and not continued.	Yes, interrupted and continued.	No, neither interrupted nor stopped.				
	**How long was the duration of the interruption of scientific studies?**						
	Less than 4 weeks	Between 4 weeks and 3 months	Between 3 and 6 months	More than 6 months			
	**Which restrictions occurred during scientific research?** *(multiple answers possible)*						
	Strict hygiene measures	Individual training	Negative COVID-test needed for participants and investigators	There were no restrictions	Other		
	**Did the COVID-19 pandemic bring concrete challenges regarding the conduction of scientific studies? Please name them.** *(optional)*						
	Free-text response						
**Q10**	**Do you want to share your feedback and opinions with us? Please let us know.** *(optional)*						
	Free-text response						

TABLE S2 Quantitative and qualitative questions within Survey 2.0 (2023).

**Q1**	**In which phase during childhood anti-cancer treatment are you offering an exercise program?** *(multiple answers possible)*								
	Acute therapy	Maintenance therapy	Follow-up care	In-patient rehabilitation facility	Other				
**Q2**	**What are you offering exactly?** *(question for each phase)*								
	Free-text answers								
**Q3**	**Can all offers within the exercise program at your site be realized like before COVID-19 pandemic without any restrictions?**								
	Yes	No							
**Q4**	**The following restrictions are still present within the exercise program during acute therapy, maintenance therapy and stationary rehabilitation.** *(individual answer for each setting separately; multiple answers possible)*								
	Program is only conducted with selected participants	Program is only conducted at selected locations	Program is only conducted with selected equipment.	Program is only conducted without any physical contact	Program is only conducted considering common hygiene measures (e.g., face mask)	Other			
**Q5**	**The following restrictions are still present within the exercise program during follow-up care.** *(multiple answers possible)*								
	Internal offers are not yet conducted (by internal staff)	External offers are not yet conducted (external staff outside the rehabilitation clinic)	Group size was reduced	Accompanied integration into sport structures (physical education at school, sports clubs, leisure-time activities) is not yet conducted	Program is only conducted considering common hygiene measures (e.g., face mask)	Other			
**Q6**	**You stated restrictions within <u>internal</u> offers during follow-up care. Which offers are affected?** *(multiple answers possible)*								
	Exercise group sessions	Exercise days	Other						
**Q7**	**You stated restrictions within <u>external</u> offers during follow-up care. Which offers are affected?** *(multiple answers possible)*								
	Exercise group sessions	Exercise days	Other						
**Q8**	**Which approaches which have been developed during COVID-19 pandemic to maintain the exercise program are maintained during acute therapy, maintenance therapy, follow-up care and in-patient rehabilitation?** *(individual answer for each setting separately) (multiple answers possible)*								
	Online training (live, with a trainer)	On-demand videos (autonomous)	Individual training	Borrowing of equipment for autonomous training	Training schedule for in-hospital exercise or at home	Outdoor training (considering the weather situation)	Consulting/motivation via telephone at home	None of those	Other
**Q7**	**Which of these approaches should be maintained for the future according to your opinion?** *(multiple answers possible)*								
	Online training (live, with a trainer)	On-demand videos (autonomous)	Individual training	Borrowing equipment for autonomous training	Training schedule for in-hospital exercise or at home	Outdoor training (considering the weather situation)	Consultants/motivation via telephone at home	None of those	Other
**Q8**	**In retrospective: How big was the impact of the following factors regarding the maintenance of the exercise offers?** *(multiple answers possible)* No impact – slight impact – medium impact – strong impact – very strong impact								
	Rules and hygiene measures within the clinics and institutions offering the program	Rules of the states or the whole country	Lack of funding	Lack of staff due to COVID-19 infections	The staff's concern to be infected by COVID-19	The participants' concern to be infected by COVID-19			
**Q9**	**What do you consider as positive aspects as exercise professional and pandemic-related restrictions regarding concepts and conduction of exercise programs in different settings?** *(optional)*								
	Free-text answers								
**Q10**	**Do you want to share your feedback and opinions with us? Please let us know.** *(optional)*								
	Free-text answers								
